# Current Advances in Imaging Diagnosis of Bacterial Infectious Diseases: From Structural to Nano Imaging Modalities

**DOI:** 10.3390/biomedicines14102329

**Published:** 2026-10-13

**Authors:** Arvin Bahreini, Alie Ametova, Nafisa Ganieva, Olim Khusenov, Raheem Rahmati, Marzeh Ramezani Farani, Mohadese Hematian, Iraj Alipourfard

**Affiliations:** 1 Department of Electrical and Computer Engineering, University of Rochester, Rochester, NY 14627, USA; 2 Department of Medical Radiology, Samarkand State Medical University, Samarkand, Uzbekistan; 3 Department of Faculty and Hospital Therapy No. 1, Rheumatology and Occupational Diseases, Tashkent State Medical University, Tashkent, Uzbekistan; 4 Department of Internal Medicine in Family Medicine, Bukhara State Medical Institute Named After Abu Ali ibn Sino, Bukhara, Uzbekistan; 5 Faculty of Medicine, Shahrekord University of Medical Sciences, Shahrekord, Iran; 6 Department of Biological Sciences and Bioengineering, Nano Bio High-Tech Materials Research Center, Inha University, 100 Inha-ro, Michuhol-gu, Incheon 22212, Republic of Korea; 7 Department of Pharmacotherapy and Pharmaceutical Care, Faculty of Pharmacy, Medical University of Warsaw, 02-097 Warsaw, Poland

**Keywords:** bacterial infections, infectious disease diagnosis, PET/CT, SPECT, radiotracers, nanoimaging

## Abstract

Infectious diseases significantly contribute to mortality and pose a growing public health challenge. Early diagnosis and treatment of acute bacterial infections are critical but often hindered by delayed or inadequate treatment. Rapid and accurate diagnostic tools are essential to manage such infections effectively. Direct imaging of infections has emerged as a promising diagnostic approach. Traditional methods rely on microscopy and bacterial culture from clinical samples, but bacterial growth can be slow and difficult, delaying diagnosis and worsening patient outcomes. This manuscript provides an overview of diagnostic methods for infectious diseases, emphasizing modern imaging techniques. This is a narrative review based on a literature search using databases like PubMed, Scopus, and Web of Science, with keywords related to infectious diseases and imaging methods. It discusses recent trials in imaging strategies for diagnosing infections and explores their potential applications. Evaluating innovative tracers for imaging infections requires considering factors such as infection models, pathogen strains, imaging settings, and tracer uptake in infected tissues. Correlating tracer uptake with tissue data and pathogen viability cultured from tissues is crucial. This review highlights the need for advanced imaging tools to improve diagnosis and patient outcomes in infectious disease management.

## 1. Introduction

Microbial infections and their complications are undoubtedly among the leading causes of death worldwide, particularly in developing countries [1,2]. Microbes, including parasites, bacteria, fungi, and viruses, cause infections that range from mild and self-limiting to severely debilitating diseases that can result in death and significant morbidity among affected populations [3,4]. The severity of bacterial infections varies; some are self-limiting, while others are life-threatening, leading to serious complications and substantial mortality [5,6]. Many infections can be effectively treated if accurately diagnosed in their early stages. Most infections are curable when the infectious process is still reversible and major damage has not yet occurred [5,6]. Although certain signs and symptoms, such as fever, pain, loss of appetite, and general weakness, are considered clinical features of infectious diseases, accurate and specific tests are essential for definitive diagnosis [7,8]. The diagnosis of microbial infections can be performed using various methods that include culture, microscopy, serology, and molecular techniques such as matrix-assisted laser desorption ionization-time of flight-mass spectrometry (MALDI-ToF-MS), PCR, and real-time PCR [9,10]. Radiological evidence plays a crucial role in diagnosing and treating microbial infections, especially those caused by specific microorganisms such as Mycobacterium tuberculosis and the novel Severe Acute Respiratory Syndrome Coronavirus-2 (SARS-CoV-2) [11,12]. While there are advantages to using radiology for diagnosing infectious diseases—such as the ability to directly visualize the infected site and monitor treatment progress—the main disadvantage is the reliance on advanced and expensive equipment [11,12]. Furthermore, imaging techniques like computed tomography (CT) and ultrasound can visualize microbial infections and inflammation of internal organs that lead to fluid accumulation and abscess formation [13,14]. Imaging tests are often employed to aid in diagnosing infections in acutely ill patients. Structural imaging modalities include ultrasound, X-ray, CT, and magnetic resonance imaging (MRI). These methods can detect abnormal collections of tissue or body fluids commonly associated with bacterial infections. For instance, point-of-care ultrasound can serve as an effective method for identifying the source of infection early in emergency settings [15,16]. A chest X-ray is typically used to detect consolidations, ventilated parts of the lungs that fill with tissue and fluid, while CT and MRI can demonstrate the presence of abscesses and inflammation. These structural approaches can be complemented by nuclear medicine techniques (SPECT and PET), which represent a type of molecular imaging used to study biochemical and physiological abnormalities. SPECT and PET are particularly useful when tissue samples are unclear or difficult to obtain [15,16]. MRI and CT primarily identify infections at later stages when significant anatomical changes have already occurred. This limitation can delay diagnosis and treatment—which is especially critical in acute cases where timely intervention is essential. In contrast, nuclear medicine techniques like SPECT and PET offer distinct advantages by utilizing routine tracers capable of detecting affected sites regardless of whether the underlying condition is an infection or sterile inflammation. This capability enables earlier identification of pathological processes that could lead to more timely management. Moreover, while structural imaging methods such as ultrasound, X-ray, CT, and MRI effectively visualize fluid accumulation and abscess formation associated with infections, they may lack sensitivity in early detection of inflammatory processes. For example, point-of-care ultrasound can quickly identify infection sources in emergency settings but may miss subtle changes detectable by SPECT or PET. Together, these observations indicate that structural and functional imaging are complementary rather than interchangeable: structural techniques remain limited in detecting early-stage infection, while SPECT and PET add biochemical and physiological context, particularly when tissue biopsy for microbiological culture is difficult to obtain. Because infection-severity assessment still relies on combining these imaging findings with clinical judgment rather than on imaging alone, the sections that follow examine each modality class in turn, focusing on where the evidence for pathogen-specific detection is strongest and where it remains preliminary. Beyond nuclear medicine, label-free vibrational spectroscopic techniques, most notably Raman spectroscopy, have likewise demonstrated broad value across biomedical diagnostics. For instance, non-plasmonic surface-enhanced Raman scattering biosensor templates have recently been engineered for point-of-care detection of cancer biomarkers such as the epidermal growth factor receptor, illustrating the wider diagnostic potential of Raman-based platforms that this review later examines specifically in the context of bacterial infection imaging [17]. In this review article, we consider various aspects: imaging methods used for diagnosing bacterial infections, anatomical or morphological imaging techniques, functional imaging approaches, nanoimaging technologies, as well as future perspectives.

Although recent literature has addressed individual facets of infection imaging, for instance, a 2024 review cataloging radiopharmaceutical chemistry specifically for bacterial infection tracers [18], there remains a lack of a single, integrative account spanning the full continuum from conventional anatomical imaging through nuclear/functional techniques to emerging nanoimaging platforms, while consistently framing each modality against the central diagnostic challenge of distinguishing true bacterial infection from sterile inflammation. This review addresses that gap by providing a unified, comparative framework across modality classes, situating recent pathogen-specific tracer and nanoprobe developments within their preclinical and clinical performance context, and outlining the practical and translational barriers each modality must overcome to reach routine practice.

Highlights
  - Traditional anatomical imaging (CT/MRI) often fails to differentiate between infection and sterile inflammation, leading to delayed diagnoses.
  - Functional imaging modalities (PET/SPECT) utilizing pathogen-specific tracers offer superior sensitivity and specificity for the early detection of bacterial infections.
  - Nanotechnology-based imaging probes enable the dual capability of real-time bacterial visualization and image-guided targeted therapy.

## 2. Imaging and Radiological Methods in Diagnoses

Diagnostic imaging procedures have become integral to modern medicine. Well-known procedures such as ultrasound, endoscopy, diagnostic X-ray imaging (DXA), computed tomography (CT), and magnetic resonance imaging (MRI) are each performed millions of times annually worldwide. Although these methods have been established for several decades, further developments that improve diagnostic evaluation continue to emerge. Additionally, there are lesser-known or entirely new imaging procedures along with hybrid techniques that combine advantages from different modalities. Table 1 provides an overview of currently important image processing procedures in medical diagnostics, along with their areas of application.

### 2.1. Imaging Methods Specified for the Diagnosis of Bacterial Infections

The identification of systemic infections through imaging techniques relies on monitoring morphological changes using ultrasound, radiography, magnetic resonance imaging (MRI), and computed tomography (CT) [43,44]. CT and MRI primarily provide valuable data for detecting infections in organs and the musculoskeletal system. However, the information obtained from these imaging modalities is often only available at late stages of infection and may be affected by morphological distortions caused by postoperative changes, scarring, and the presence of foreign bodies [43,44]. Furthermore, most imaging techniques are limited by infection-induced inflammation, which complicates the differentiation between tumors, abscesses, and causative pathogens [45,46]. To complement the morphological imaging techniques described above, radiolabeled markers such as proteins and cells can be utilized to visualize the infection process through positron emission tomography (PET) or single photon emission computed tomography (SPECT). SPECT is a powerful imaging technique that enables the visualization of biological processes in vivo. One of its promising applications is the detection of infections, where specific tracers can target various aspects of microbial presence and activity. This includes the detection of cell wall components, metabolic activity, and virulence factors of pathogens. SPECT tracers for infection detection are Cell Wall Targeting Tracers: Many bacteria have unique components in their cell walls that can be targeted by specific tracers [47,48]. For instance, peptidoglycan, a major component of bacterial cell walls, can be targeted using radiolabeled antibodies or peptides that bind specifically to these structures [47,48]. This allows for the visualization of bacterial infections in tissues. **B:** Metabolic Activity Tracers: Tracers that reflect metabolic activity can provide insights into the viability and proliferation of pathogens. Radiolabeled substrates that are preferentially taken up by metabolically active cells can be used. For example, tracers like ^18^F-FDG (fluorodeoxyglucose) are commonly used in PET imaging to assess areas of infection based on increased glucose metabolism, which is often seen in active infections [49,50]. It should be clarified that fluorine-18, the radionuclide in FDG, is a positron emitter, and clinical FDG imaging is therefore performed with dedicated PET systems that detect coincident 511-keV photon pairs using a full or partial detector ring. Historically, so-called “FDG-SPECT” was investigated as a lower-cost alternative using specially configured gamma cameras, either by fitting conventional SPECT cameras with ultra-high-energy collimators to detect single 511-keV photons directly, or by operating dual-head gamma cameras in coincidence mode, counting only photon pairs detected simultaneously at 180° from one another [51]. These approaches do not represent conventional SPECT acquisition, require non-standard hardware modifications, and are not the current standard method for ^18^F-FDG infection imaging; they have been largely superseded by the now-widespread clinical availability of dedicated PET/CT, owing to their comparatively lower sensitivity, poorer spatial resolution, and lower target-to-background ratios relative to dedicated PET [52]. Separately, and unrelated to this historical gamma-camera approach, structurally distinct glucose analogs labeled with the gamma-emitting radionuclide technetium-99m (e.g., ^99m^Tc-labeled deoxyglucose/ethylenedicysteine-deoxyglucose derivatives) have also been synthesized to mimic the biodistribution and glucose-metabolism-dependent uptake of ^18^F-FDG for conventional SPECT-based infection and tumor imaging [53]. **C;** Virulence Factor Targeting: Some tracers can be designed to bind to virulence factors produced by pathogens. For example, certain peptides or antibodies can be labeled with a radioactive isotope and used to target specific toxins or enzymes that are indicative of pathogenicity. This approach can help differentiate between benign and virulent strains of bacteria [54]. **D;** UBI Peptides and Antimicrobial Peptides (AMPs) UBI peptides (ubiquicidin-derived antimicrobial peptides, particularly the fragment UBI(29-41), originally isolated from mouse macrophages and unrelated to the eukaryotic ubiquitin protein despite the similar name [55]) and other antimicrobial peptides (AMPs) represent a novel class of compounds that can be utilized as SPECT tracers for infection detection. These peptides have several advantageous properties [56,57]. UBI peptides can be engineered to bind specifically to bacterial cell wall components or other markers of infection, enhancing the specificity of imaging. Many AMPs possess inherent antimicrobial properties, which can help in reducing the bacterial load while simultaneously providing imaging capabilities. AMPs often have a rapid clearance from the bloodstream, which can improve the signal-to-noise ratio in imaging studies [56,57]. The use of SPECT tracers targeting specific aspects of infection holds great promise for improving diagnostic accuracy and treatment monitoring. Potential applications include:

- **Early Detection**: SPECT imaging with specific tracers can facilitate the early detection of infections, allowing for timely intervention;
- **Treatment Monitoring**: By assessing changes in tracer uptake, clinicians can monitor the effectiveness of antimicrobial therapies;
- **Research**: These tracers can be invaluable in research settings for studying the dynamics of infections and the host response.

These SPECT tracer strategies—targeting cell wall components, metabolic activity, and virulence factors, with UBI peptides and other AMPs as a further avenue for improving specificity—are summarized, along with their targets, mechanisms, and examples, in Table 2.

**Figure 1:**
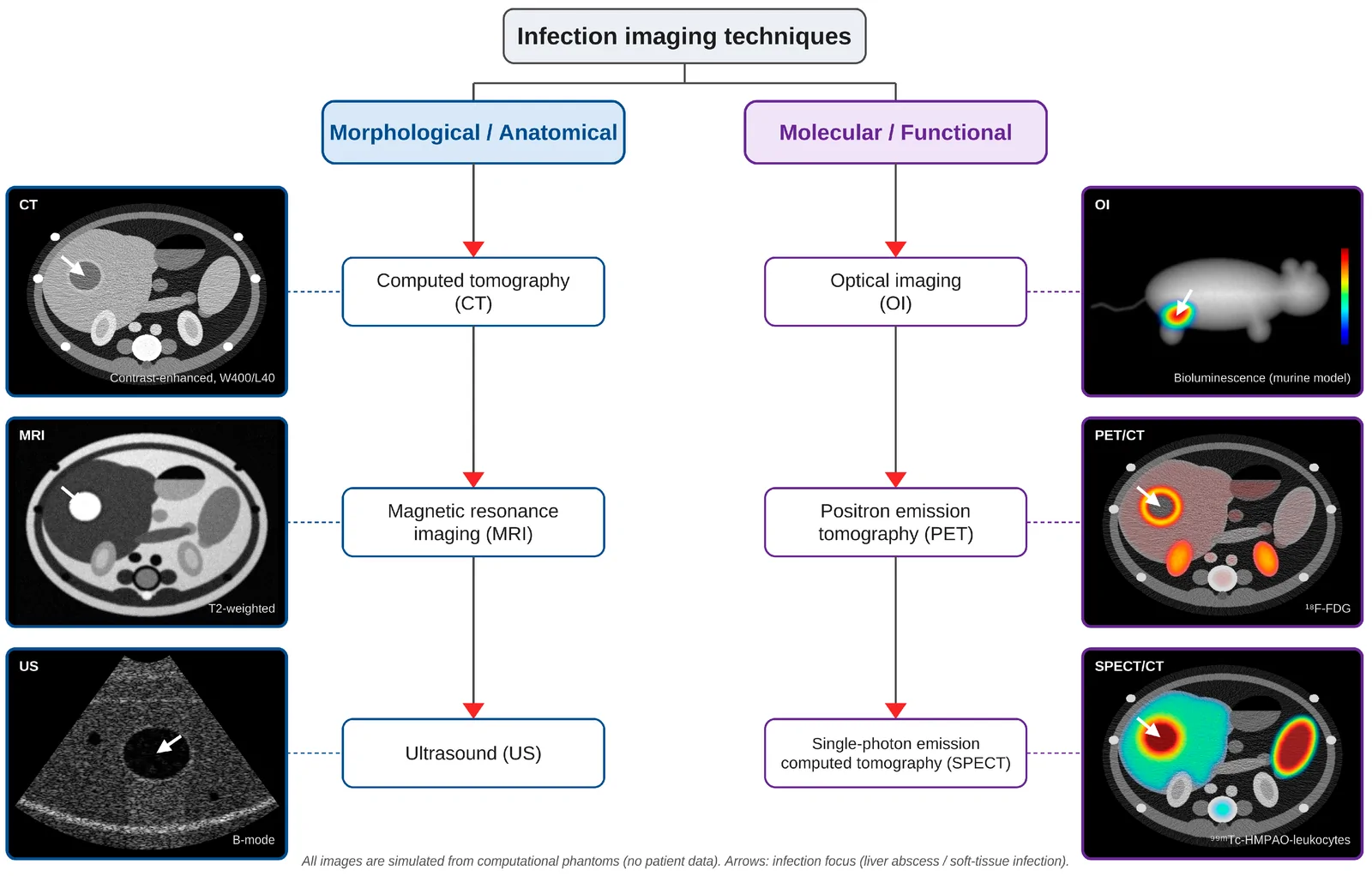
Overview of imaging approaches used in the diagnosis of infection. Morphological/anatomical modalities (computed tomography, magnetic resonance imaging and ultrasound) depict structural changes, whereas molecular/functional modalities (optical imaging, positron emission tomography and single-photon emission computed tomography) depict biological or metabolic activity at the site of infection. Representative images show a hepatic abscess (CT, T2-weighted MRI, B-mode ultrasound, ^18^F-FDG PET/CT and ^99m^Tc-HMPAO-labelled leukocyte SPECT/CT) and a bioluminescent soft-tissue infection in a murine model (optical imaging); arrows indicate the infection focus. All images were simulated by the authors from computational phantoms and do not represent patient or animal data.

Imaging studies can be categorized into two main types: anatomical studies and functional studies [58,59]. Positron emission tomography (PET) is a nuclear medicine procedure that measures the metabolic activity of cells in body tissues. PET effectively combines nuclear medicine with biochemical analysis [60,61]. A single-photon emission computed tomography (SPECT) scan allows physicians to analyze the organs, tissues, and bones of the body. As a type of nuclear imaging test, SPECT utilizes a radioactive substance and a specialized camera to create three-dimensional images [61,62]. Figure 1 illustrates various imaging approaches used in infection diagnosis.

Assessing patients who may be infected poses significant challenges. Imaging techniques designed to locate or identify the site of infection are often limited by the nonspecific results produced by traditional imaging modalities. Available imaging techniques may not optimize treatment responses or determine whether antibiotics are effectively reaching the site of infection. PET is a method capable of detecting specific targets or expression levels of metabolic disorders using radiolabeled molecules. To address these imaging deficiencies, the development of pathogen-specific PET radiotracers is necessary. Although progress has been made in both preclinical and clinical settings, substantial challenges remain.

#### 2.1.1. Anatomical or Morphological Imaging of Bacterial Infections

As its name suggests, anatomical or morphological imaging in bacterial infections reveals structural changes in tissues or organs caused by microbial invasion and the host’s inflammatory response during infection [63,64]. X-rays, computed tomography (CT), ultrasound, and magnetic resonance imaging (MRI) are all examples of morphological imaging studies [58,65]. The term “CT” refers to a computerized X-ray technique in which a narrow beam of X-rays is directed at a patient and rapidly rotated around the body, creating signals that are processed by the machine’s computer to produce cross-sectional images, or “slices.” Tomographic images provide clinicians with more detailed information than conventional X-rays [66,67,68]. Once a series of successive slices is collected by the machine’s computer, they can be digitally combined to form a three-dimensional (3D) image of the patient. This allows for easier documentation of basic structures as well as the identification of potential tumors or anomalies [66,67]. Pyogenic *Streptococcus* lung parenchyma infection is characterized by the presence of pus-forming bacteria within the lung tissue, leading to inflammation and tissue damage. This infection can result in symptoms such as cough, fever, and difficulty breathing, often necessitating prompt medical intervention. Effective treatment typically involves antibiotics and supportive care to manage respiratory function and prevent complications. The inflammatory response alters the normal density of host tissue due to pyogenic infection, resulting in changes observable on CT images. For instance, in pyogenic pneumonia, an exudate with a density similar to water replaces the air in the alveoli. Consequently, the affected regions of the lung appear gray on CT scans, whereas healthy lung parenchyma, filled with air, typically appears black. Bacterial imaging of *S. aureus* in endothelial cells facilitates visualization of bacterial interactions with the vascular endothelium, which is crucial for understanding infection mechanisms. Techniques such as fluorescence microscopy and Raman spectroscopy can reveal the localization and behavior of *S. aureus* within these cells, providing insights into its pathogenicity. This imaging approach aids in studying the inflammatory response and identifying potential therapeutic targets for infections caused by this opportunistic pathogen. An innovative Raman-based imaging technique provides high-quality false-color images to sensitively detect intracellular *S. aureus* and precisely focus on these bacteria in three dimensions (3D) within endothelial cells. Advanced techniques such as confocal microscopy and Raman spectroscopy enable visualization of *S. pneumoniae* interactions with host cells and tissues, revealing insights into its virulence and colonization mechanisms. These imaging methods contribute to the development of targeted therapies and vaccines by elucidating the pathogen’s behavior within the host environment. A radiographic method has been developed for detecting community-acquired pneumonia (CAP), utilizing procalcitonin (PCT) and highly sensitive C-reactive protein (hsCRP) detected by radiographs. Pulmonary bacterial secondary infections and co-infections with SARS-CoV-2 are relatively uncommon. Standard CT was employed to detect pulmonary bacterial infections such as *S. pneumoniae* and *E. faecalis* in adult patients hospitalized for COVID-19. Additionally, CT techniques were utilized in emergency departments to assess soft tissue and skin extremity infections. A gadolinium (Gd)-based MRI nanoaggregate probe termed GFV, composed of a self-assembling peptide (FFYEGK) combined with the antibiotic vancomycin (Van), co-modifying the gadolinium chelate, has been developed for identifying *S. aureus* infections in vivo and monitoring treatment of *S. aureus*-infected myositis using daptomycin (Dap) as a model antibiotic. For managing bacterial meningitis in infants, brain MRI serves as a conventional and sensitive method that confirms abnormal changes in the brain. While vancomycin is a critical antibiotic for treating infections caused by Gram-positive bacteria, its utility in imaging is restricted to strains that are susceptible to it. This limitation poses a significant challenge in clinical settings, especially given the rising prevalence of vancomycin-resistant *Staphylococcus aureus* (VRSA). Despite this susceptibility-dependent limitation, novel probes such as GFV offer a route to improved detection and management of *S. aureus* infections, including those caused by resistant strains. MRI has been developed as an imaging modality for evaluating musculoskeletal (MSK) joints, soft tissue, and bone infections. Skin and soft tissue infections (SSTIs), such as wound infections, abscesses, and cellulitis, commonly occur in emergency departments (EDs) and primary care settings. Increasing evidence suggests that ultrasound (US) can enhance diagnostic accuracy for SSTIs and may influence treatment decisions [69,70]. Using ultrasound, clinicians can detect fluid accumulation, edema, or soft tissue cords indicative of inflammation. Signs of more serious infection include perifascial fluid and subcutaneous emphysema, which may also be revealed through US imaging [69,70]. Gallbladder perforation is a life-threatening complication of acute cholecystitis that is often challenging to diagnose in its early stages. Standard X-rays and laboratory tests are unreliable for recognizing patients with this complication. In contrast, one study found that biliary ultrasound accurately diagnosed preoperative pericholecystic abscesses in patients with acute cholecystitis; ultrasound findings were consistent across all three patients studied. The findings indicated that the ultrasound frequency used in microbial diagnosis significantly impacts the survival rate of bacteria causing periprosthetic joint infection (PJI). In one research study involving pediatric populations, US techniques were applied to determine peritonsillar abscess (PTA). Results revealed that over 15% of patients who underwent US evaluation for PTA required secondary imaging (Table 3). Raman-based imaging techniques have emerged as powerful tools for the anatomical and morphological characterization of bacterial infections. These approaches leverage unique vibrational signatures from molecular bonds within bacterial cells, allowing for noninvasive and label-free analysis [71,72]. Recent work has continued to advance the underlying sensing technology and its clinical applicability: engineered two-dimensional-material/nanoparticle hybrid substrates have been shown to substantially improve the signal-to-noise ratio of surface-enhanced Raman spectroscopy (SERS) measurements [73], SERS combined with machine-learning classification has been used to rapidly and accurately discriminate viral proteins such as the SARS-CoV-2 receptor-binding domain, illustrating the broader pathogen-identification potential of this approach beyond bacteria [74], and a 2026 review specifically traces the translation of SERS-based bacterial detection from nanomaterial substrates toward integrated, point-of-care-ready clinical platforms [75]. Taken together, these label-free spectroscopic approaches continue to mature as tools for the anatomical and morphological characterization of bacterial infections [71,72]. As shown in Table 3, a wide range of bacteria can cause different types of infections; however, morphological imaging methods are often unable to detect and differentiate between bacterial strains.

#### 2.1.2. Imaging Diagnosis of COVID-19 and Its Distinction from Bacterial Pulmonary Infection

Coronavirus disease 2019 (COVID-19), caused by SARS-CoV-2, offers an instructive comparison for the central theme of this review, since it illustrates how structural imaging alone can struggle to separate viral, bacterial, and mixed pulmonary infections. Chest CT remains a key adjunct to reverse transcription-polymerase chain reaction (RT-PCR) testing for COVID-19 pneumonia, typically showing bilateral, peripherally distributed ground-glass opacities (GGOs), often with a crazy-paving pattern, whereas bacterial pneumonia more frequently presents as unilateral, lobar consolidation with air bronchograms, pleural effusion, and lymphadenopathy. A 2023 study identified the arched bridge and vacuole signs as CT features that help distinguish COVID-19 pneumonia from both influenza and bacterial pneumonia, supporting earlier, non-invasive triage decisions [90]. This distinction is clinically important given that bacterial co-infection or secondary bacterial pneumonia complicates a subset of COVID-19 admissions and is associated with worse outcomes, meaning imaging findings must be interpreted alongside clinical and microbiological data rather than in isolation, consistent with the broader argument of this review.

Beyond conventional CT, nanotechnology-based diagnostic platforms have been explored as faster, potential point-of-care alternatives or complements to RT-PCR and CT for detecting SARS-CoV-2, including nanoparticle-based biosensors, lateral-flow immunoassays, and nano-enhanced molecular assays, which have reported diagnostic performance comparable to RT-PCR and CT in early evaluations [91]. While these nano-based methods target the virus itself rather than bacterial pathogens, they exemplify the broader trend toward nanoimaging and nanodiagnostic platforms discussed later in this review (Section 3) and further reinforce the rationale for a diagnostic approach that integrates structural imaging with molecular and nanoscale detection strategies.

### 2.2. Functional Imaging Approaches

**Figure 2:**
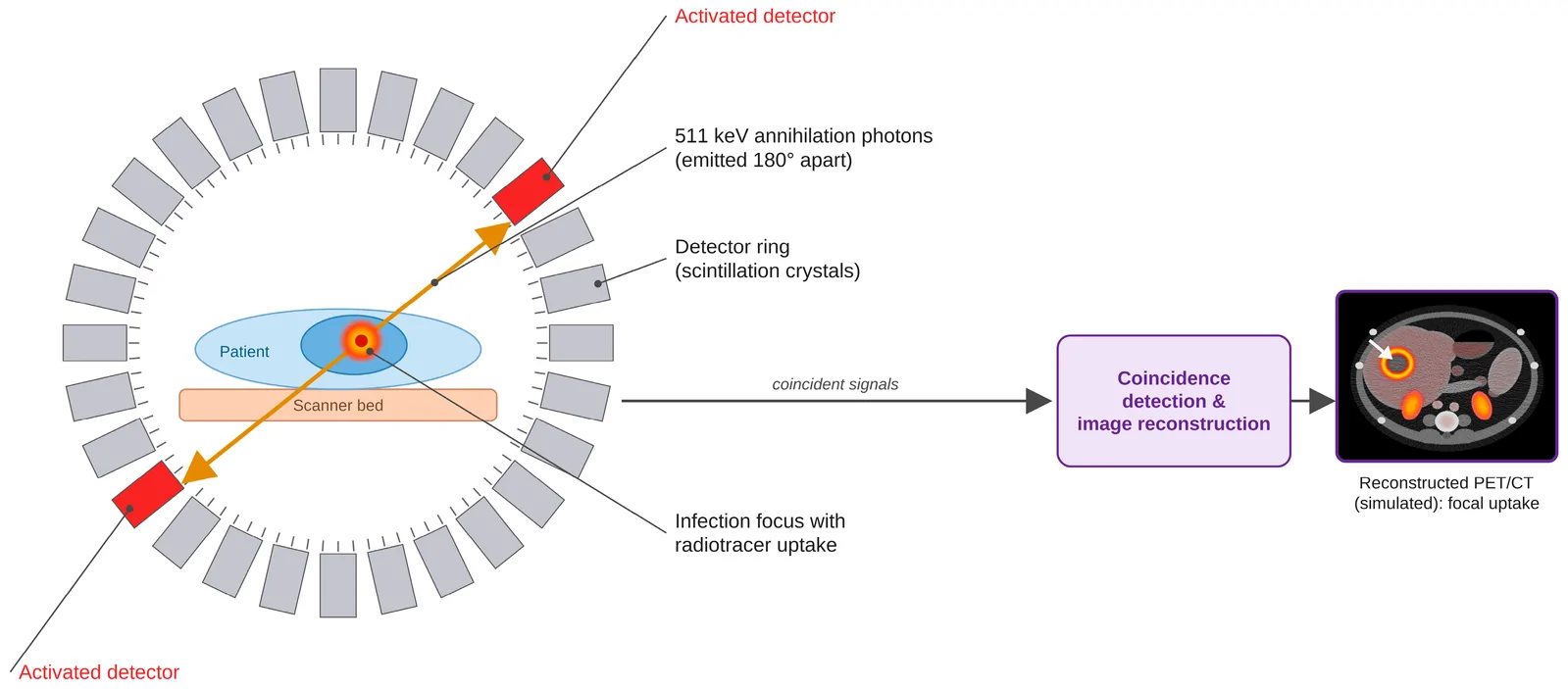
Schematic representation of the coincidence-detection principle of positron emission tomography (PET). A positron emitted by the radiotracer accumulated at the infection focus annihilates with an electron, producing two 511 keV photons that travel in opposite directions (180° apart) and are registered simultaneously by a pair of detectors in the surrounding ring. Coincidence events from many photon pairs are processed to reconstruct an image showing focal tracer uptake. Original schematic created by the authors; the reconstructed PET/CT image was simulated from a computational phantom and does not represent patient data.

Functional imaging approaches, such as nuclear medicine studies, utilize small amounts of radioactive materials or tracers [58,65]. These tracers are typically taken up directly by tissues, organs, and cells or attached to natural substances that are transferred to the area of interest [65,92]. For example, radiolabeled diphosphonates are incorporated directly into bone tissue. Indium-111 can be bound within the cells of white blood cells and is used to monitor the accumulation of these cells at the site of infection. Other functional imaging tests employed to localize infections include ^18^F fluorodeoxyglucose (FDG) and positron emission tomography (PET) (Figure 2). Traditional imaging techniques for identifying and treating bacterial infections are relatively sensitive but often exhibit low specificity. FIAU (1-(2-deoxy-2-fluoro-β-D-arabinofuranosyl)-5-iodouracil) is a nucleoside analog that circulates freely between the intracellular and extracellular environments. This compound becomes trapped in the cellular space when phosphorylated by thymidine kinase (TK). Therefore, if FIAU is labeled with a specific radioactive isotope, cells expressing thymidine kinase can be identified using SPECT or PET methods. In this context, an imaging procedure was developed in a study to evaluate the potential use of [^124^I] FIAU in infections of the musculoskeletal system [93]. Biofilm-associated infections, such as prosthetic joint infections and chronic osteomyelitis, are particularly challenging to treat because biofilms protect bacteria from the host’s immune response, allowing subpopulations of bacteria residing within biofilms to become dormant [94,95]. Biofilm penetration tracers are of interest for detecting persistently and chronically infected tissues and implants, serving as alternatives to tracers that cannot penetrate biofilms and, therefore, cannot effectively target bacteria [96,97]. The identification of innovative fluorescent probes for biofilm imaging will significantly facilitate the diagnosis of pathogenic bacterial infections [96,97].

**Figure 3:**
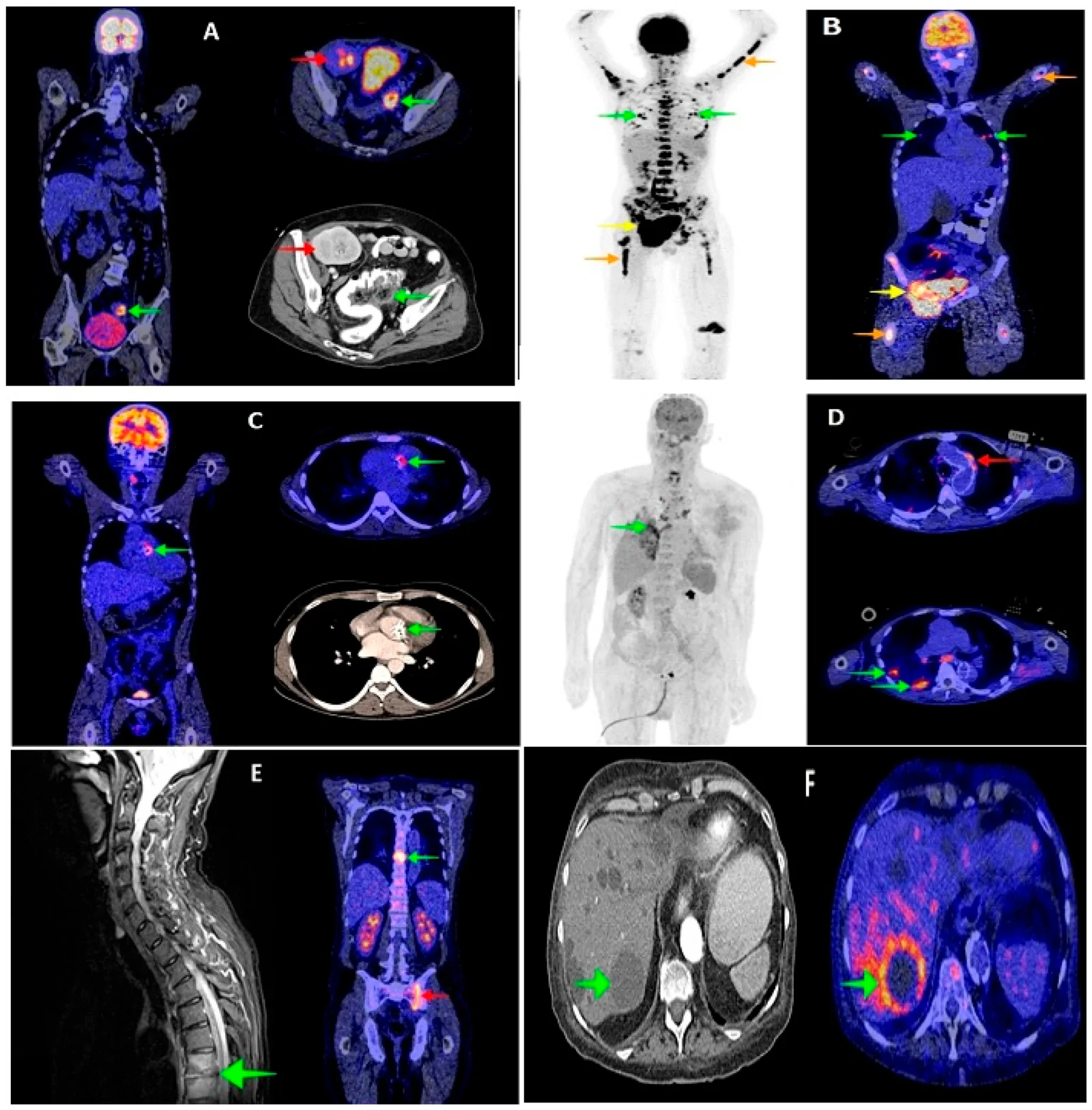
FDG-PET/CT technique for patients (**A**), multiple potential infection foci; (**B**), fever of unknown origin; (**C**), infective endocarditis; (**D**), vascular graft infection; (**E**), spondylodiscitis and (**F**), cyst infection were presented from (**A**–**F**)**, respectively,** adapted from Ref [115]. Microbiological correlation for these illustrative panels follows the original source [115]. Explicit culture confirmation is reported in the present text only for the cyst infection case (culture-positive *Escherichia coli*, as described above); for the remaining panels (**A**–**E**), the original report characterized the cases using composite clinical, radiological, and laboratory criteria rather than explicit per-panel culture confirmation. This distinction is noted here because, as emphasized throughout this review, imaging findings, however characteristic, should be interpreted alongside microbiological and tissue-based confirmation rather than in isolation, since FDG-PET/CT cannot itself discriminate infection from sterile inflammation.

A novel fluorescent probe, CDy11 (designated compound Yellow 11), targets amyloid in *Pseudomonas aeruginosa* biofilm matrices through a diversity-directed fluorescent library approach (DOFLA). CDy11 has also been demonstrated in in vivo imaging of *P. aeruginosa* in implants and mouse models of corneal infection [98]. Additionally, a zinc-dipicolylamine analog (Zn-DPA)-biotin complex, formed by non-covalently linking 111In-DOTA-biotin with streptavidin (SA), has been advanced for improved visualization upon bacterial absorption using SPECT-CT imaging [99]. Findings indicate that PET with 2-deoxy-2-[^18^F]fluoro-D-sorbitol (^18^F-FDS) can sensitively detect *Enterobacterales* infections in murine models [100]. The uptake of ^18^F-FDS by bacteria occurs through a metabolically conserved sorbitol-specific pathway, and rapid in vitro uptake of ^18^F-FDS is observed in clinical strains, including multidrug-resistant (MDR) isolates. Whole-body ^18^F-FDS-PET/computed tomography (CT) in patients with microbiologically confirmed Enterobacterales infections or other disease states demonstrates that ^18^F-FDS-PET/CT is safe and can rapidly detect and localize drug-induced Enterobacterales infections. This technique successfully identified MDR strains and differentiated them from sterile inflammatory and cancerous lesions [101]. The identification of cardiac implantable electronic device (CIED) infections is noteworthy due to their variable presentations. The importance of 2-[^18^F]fluoro-2-deoxy-D-glucose (^18^F-FDG) PET/CT in detecting CIED infections has recently advanced [102]. Results indicate that ^18^F-FDG PET/CT plays an increasingly important role in diagnosing and monitoring complex infections in immunocompromised, high-risk patients. In this study, FDG-PET/CT was utilized to evaluate opportunistic infections and complex invasive fungal diseases in patients with acute leukemia prior to allogeneic hematopoietic cell transplantation [103]. Although vascular graft and endovascular graft infections (VGEIs) are uncommon, diagnostic complications can have serious consequences, including mortality rates ranging from 25% to 88% [104,105]. Additionally, ^18^F-FDG PET/CT was employed to identify vascular graft and endograft infections (VGEIs). PET imaging of Staphylococcus aureus infections was conducted using a nitroprodrug analog of 2-[^18^F] F-p-aminobenzoic acid. These results suggest that 2-[^18^F]F-ENB and 2-[^18^F]F-NB may be translated to humans as rapid, noninvasive diagnostic tools for identifying and localizing *S. aureus* infections [106]. The biodistribution and radioactive synthesis of ^18^F-linezolid were planned using a PET method for diagnosing mice infected with Mycobacterium tuberculosis. Following intravenous injection, ^18^F-linezolid promptly circulated to all organs and effectively reached the *M. tuberculosis*-infected lungs. Drug biodistribution studies utilizing PET can provide unbiased in situ drug quantities and may enhance efforts to optimize antibiotic dosing strategies [107]. In patients with an unknown source of infection or multiple potential infection foci, FDG-PET/CT has proven valuable for diagnosing the infection focus, thereby enabling more targeted treatment [108]. For instance, a hospitalized patient presenting with prolonged fever (3 weeks), general malaise, and weight loss underwent FDG-PET/CT. Fusion images from FDG-PET/CT and coronal maximum intensity projection revealed a large FDG-avid mass in the right minor pelvis, multiple lung metastases, and focal FDG uptake in the bone marrow [109]. Infective endocarditis is an endocardial infection associated with high morbidity and mortality [110,111]. A patient exhibiting general malaise, fever, petechiae, and a pneumococcal-positive blood culture underwent functional imaging over one week. Coronal and axial fused FDG-PET/CT demonstrated FDG avidity in the aortic valve prosthesis, suggesting infection without additional sources identified. The aortic valve was visualized on full-dose chest CT; it was subsequently surgically replaced, resulting in patient recovery [112]. Approximately 1% to 5% of vascular graft patients develop vascular graft infections [113,114]. These infections can occur immediately following vascular graft implantation (within two months post-surgery), classified as early infections, or more than two months after surgery as late infections [114]. An elderly man with vascular grafts in the abdominal aorta, aortic arch, and left common iliac artery was admitted to the hospital with a fever of 38.5 degrees Celsius and a positive blood culture for *Escherichia coli*. Maximum intensity coronal FDG-PET projection revealed increased FDG uptake in the right lower lung lobe alongside multiple metabolically active mediastinal and paratracheal lymph nodes suggestive of infection [115]. Although spondylodiscitis is relatively rare, occurring at an incidence of approximately 1:100,000 to 1:250,000 per year, its incidence is rising due to increased life expectancy, risk factors, and comorbidities [116]. The average length of hospital stay for affected patients ranges from 30 to 57 days, with reported in-hospital mortality rates between 2% and 17%. In these cases, FDG-PET/CT is frequently employed to evaluate suspected spondylodiscitis. This imaging modality may also demonstrate greater sensitivity for early spondylodiscitis compared to MRI since it detects increased glucose uptake associated with infection independent of anatomical changes [115]. Functional imaging was performed on an elderly woman presenting with general malaise, night sweats, and positive cultures for *Escherichia coli*. Fused axial FDG-PET/CT confirmed increased FDG uptake within a large cyst located in liver segment VII, suggestive of infection [115]. Figure 3 illustrates the FDG-PET/CT technique applied to patients with multiple potential infection foci including fever of unknown origin, infective endocarditis, vascular graft infection, spondylodiscitis, and cyst infection presented from panels A to F.

Bioluminescence imaging (BLI) is a phenomenon where organisms produce light through a chemical reaction. Luminescent bacteria stand out among bioluminescent organisms due to their unique characteristics. The intensity of their bioluminescence serves as a measure of their metabolic activity, providing a direct indication of how environmental factors affect cell viability [117]. In recent years, optical coherence tomography (OCT) has been demonstrated as an improved in vivo diagnostic tool for visualization and morphological characterization of OM biofilms and middle ear effusions, but it lacks specificity about the causative bacterial species [118]. Functional imaging techniques are having a major impact on therapeutic monitoring, diagnosis, treatment, and prognosis of tumor diseases. These can help improve treatment decisions, offer more personalized care for patients, and reduce healthcare costs in many situations. Functional imaging is expected to continue this movement [119,120]. This has created new goals for patient management, treatment, and clinical monitoring. At the same time, significant progress is being made in the development of highly specific imaging probes [119,120]. This facilitates the expansion of new imaging techniques, such as hybrid scanning techniques. These new targeted probes can be used in conjunction with new drugs to speed up the approval process and select the most appropriate patients for treatment [121,122]. The ability to combine the sensitivity and specificity of PET with the anatomical resolution of CT has proven invaluable and has been documented to result in improved accuracy. In the future, it should be possible to fuse two or more modalities, such as MR, SPECT, PET, or even ultrasound. Therefore, the future of hybrid imaging remains promising and is expected to be useful in the treatment of cancer patients [121,122]. Some details of functional imaging methods in the diagnosis of bacterial infections are summarized in Table 4.

As shown in Table 4, a wide range of bacteria can cause different types of infections; however, functional imaging methods are often unable to detect and differentiate between bacterial strains.

## 3. Nanoimaging of Bacterial Pathogens: Insights into Bacterial Visualization Rather than Infections

Nanotechnology is an emerging field that utilizes the manipulation of atoms and molecules to create new molecular components at the nanoscale, specifically in the nanometer range [126,127]. This innovative technology has led to the development of novel concepts in both diagnosis and treatment. In the realm of diagnostic imaging, various biomaterials are employed as imaging agents [126,127]. Nanoimaging of bacterial infections involves the application of nanotechnology to visualize and understand the dynamics of bacterial infections at the cellular and molecular levels. This approach has the potential to enhance the diagnosis and treatment of infectious diseases by providing detailed insights into the infection process.

**Figure 4:**
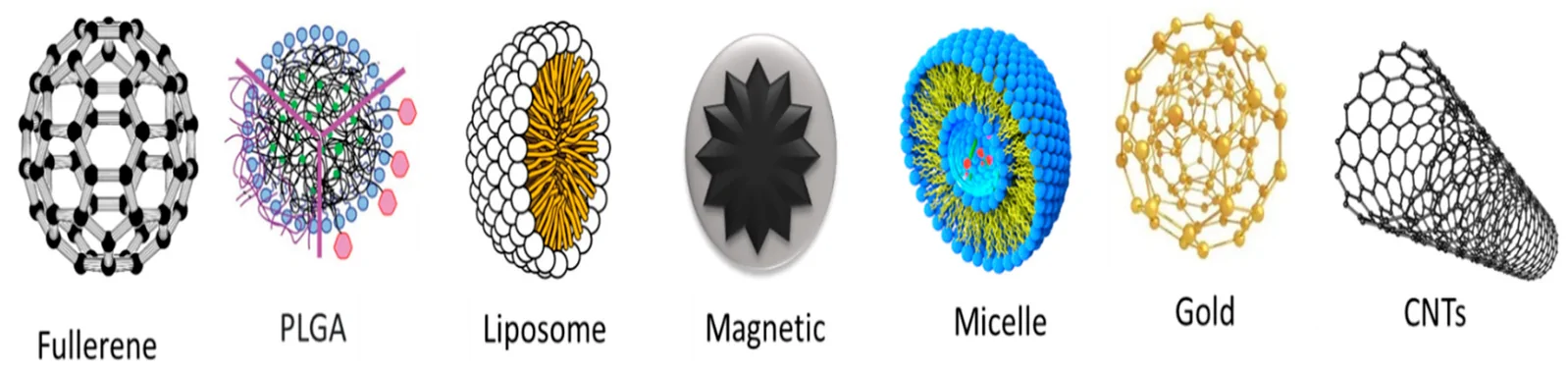
Different types of nanoparticles used in nanoimaging [an original schematic provided by the authors using Microsoft PowerPoint].

Due to their chemical and physical properties, nanoparticles can be easily modified with various ligands and conjugated to achieve site-specific ligand–molecule interactions on their surfaces, facilitating the evaluation of molecular organization [128,129] (Figure 4). The targeting mechanism of nanoparticles for bacteria represents a complex and evolving area of research, with several factors influencing their effectiveness. Understanding these mechanisms is crucial for developing targeted nanoparticle-based therapies [130,131]. One key aspect is the surface properties of nanoparticles, including their charge, hydrophobicity, and functionalization. For instance, positively charged nanoparticles often exhibit a higher affinity for negatively charged bacterial membranes, thereby enhancing their targeting capabilities [130,131]. Additionally, the size and shape of nanoparticles play significant roles; smaller nanoparticles may penetrate bacterial cells more effectively, while specific shapes can improve their ability to navigate through bacterial biofilms. Another important factor is bimolecular recognition. Some nanoparticles are engineered to mimic natural ligands or are coated with antibodies or peptides that specifically bind to bacterial surface receptors [132,133]. This targeted approach increases the selectivity of nanoparticles for particular bacterial strains, making them more effective in therapeutic applications. Once nanoparticles reach the bacterial surface, they may be taken up through endocytosis or induce membrane disruption, leading to bacterial cell death [132,133]. However, the exact pathways and mechanisms of uptake can vary depending on the type of nanoparticle and the bacterial species involved. Environmental factors such as pH, temperature, and the presence of other biomolecules also influence the behavior of nanoparticles and their interactions with bacteria. Furthermore, bacteria can develop resistance mechanisms against nanoparticles, complicating targeting strategies and necessitating a deeper understanding of these responses. These unresolved variables in nanoparticle–bacteria interactions warrant continued mechanistic study to inform more effective nanoparticle-based therapies.

Nanoimaging techniques utilize nanoparticles and nanostructures to enhance the visualization and characterization of bacterial infections at the molecular level. These techniques can be categorized into three main types: therapeutic agents, diagnostic agents, and theragnostic agents, each serving distinct roles in the management of infections. Nanoimaging represents a novel form of molecular imaging modality based on the use of various nanomaterials, including nanoparticles, nanoshells, nanorods, nanospheres, and nanostars, primarily used in biomedical and oncology imaging. The application of nanomaterials can complement any imaging technique, including computed tomography (CT), magnetic resonance imaging (MRI), positron emission tomography (PET), single-photon emission computed tomography (SPECT), ultrasound, optical imaging, and photoacoustic imaging. In addition to these diagnostic modalities, various treatment options, such as photodynamic therapies, photothermal therapies, chemotherapy, and immunotherapies, have emerged [128,129].

**Figure 5:**
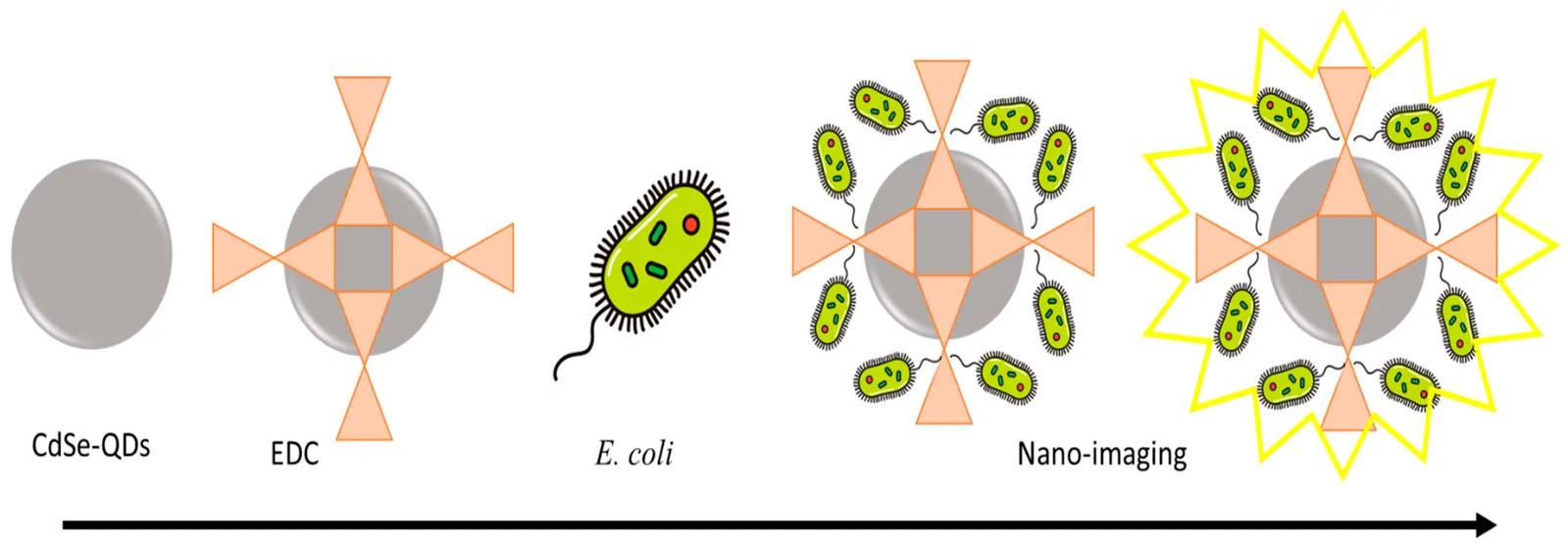
Nano-imaging of *E. coli* based on CdSe QDs. [an original schematic provided by the authors using Microsoft PowerPoint].

Engineered nanomaterials with versatile functionality have shown promise in diagnosing pathogens in food, blood, and clinical samples. The unique optical and magnetic properties of nanoscale materials have been harnessed for diagnostic purposes [134,135]. To date, various types of nanoparticles (NPs) have been studied and proven promising for the nano-diagnosis of infectious diseases [134,135]. Nanoparticles can be functionalized with specific ligands (e.g., antibodies, peptides, or aptamers) that bind to bacterial surface markers. This functionalization enhances the specificity of the nanoparticles for target bacteria, allowing for more accurate imaging. Additionally, surface coatings can be applied to improve biocompatibility, stability, and circulation time in the bloodstream. Common coatings include polyethylene glycol (PEG), which reduces immunogenicity and enhances solubility. Furthermore, nanoparticles can be loaded with antimicrobial agents and engineered to release these drugs in response to specific stimuli (e.g., pH changes or enzymatic activity) at the infection site. This dual functionality of imaging and therapy, termed theranostics, holds significant therapeutic potential. In vivo imaging allows researchers to track the distribution and localization of bacteria in real-time, providing insights into infection dynamics and treatment effectiveness. Additionally, nanoimaging can be employed in cell culture models to study bacterial interactions with host cells; nanoparticles can be introduced to infected cell cultures to visualize bacterial uptake and intracellular localization. Nanoparticle technologies based on fluorescent nanoparticles (e.g., dye-loaded nanoparticles and quantum dots), magnetic nanoparticles, and metallic nanoparticles (e.g., gold and silver nanoparticles) have been employed to image, track, and detect a variety of infectious microorganisms [134,135]. For instance, biotinylated antibodies combined with quantum dots (Qdots) were utilized for the detection of *Escherichia coli* O157:H7. In this study, fluorescence microscopy was applied for the sensitive detection of microbial cells [136]. Magnetic nanoparticles (MNPs) were also used for the rapid and sensitive fluorometry-based detection of *E. coli* O157:H7, *Salmonella typhimurium*, and *Listeria monocytogenes* in spinach, chicken, and milk samples [137]. Similarly, MNPs combined with Qdots were applied for ultra-sensitive detection of *E. coli* O157:H7 and *Salmonella enterica* in milk samples [138]. Bacteria labeled with cadmium selenide quantum dots (CdSe QDs), specifically *E. coli*, were tested on both agar nutrient substrates and groundfish substrates to observe their growth rates in different laboratory environments (Figure 5). Samples with a suitable concentration ratio of 10^7^ CFU/mL bacteria to CdSe QDs were empirically selected from samples grown on agar-containing plates [139].

Dye-doped silica nanoparticles (NPs) conjugated with antibodies have been employed for the detection of *Mycobacterium tuberculosis*. In this research, amplified signals were detectable by fluorescence microscopy within four hours [140]. The findings indicated that manganese oxide (MnO2) NPs are applicable for bacterial infection detection and therapy [141]. Methicillin-resistant *Staphylococcus aureus* infections in mice were identified using fluorescence-labeled polymeric NPs carrying vancomycin as the targeting agent [142]. Fluorescent silicon nanoparticle-based nanotheranostic agents were applied for the rapid diagnosis and treatment of bacteria-induced keratitis. The results suggest that SiNPs-Van could serve as a novel high-quality nanotheranostic agent, particularly suitable for immediate diagnosis and therapy of Gram-positive bacterial keratitis [143] (Table 5).

**Table 5:** Nanoimaging methods of pathogenic bacteria.

Pathogen	Nanomaterial	Recognition	Detection Method	Ref
*E. coli O157:H7*	Q dots	Biotinylated antibody	Fluorescence microscopy	[136]
*E. coli O157:H7*, *S. typhimurium*, *L. monocytogenes*	MNPs	Biotinylated antibody	Fluorescence microscopy	[137]
*E. coli O157:H7* and *S. enterica*	MNPs with Qdots	Biotinylated antibody	Fluorescence microscopy	[138]
*E. coli O157:H7*	Qdots	EDC/UV light	Fluorescence microscopy	[139]
*M. Tuberculosis*	Dye-doped silica NPs	Antibody	Fluorescence microscopy	[140]
Complex microorganisms	(MnO_2_) NPs	EDC/UV light	Photodynamic	[141]
*S. aureus*	Fluorescence-labeled polymeric nanoparticle (NP)	Fluorescence probe	Fluorescence	[142]
*S. aureus*	SiNPs	Fluorescence probe	Fluorescent	[143]

1-ethyl-3-(3-dimethylaminopropyl)-carbodiimide (**EDC**).

Nanoimaging of bacterial infections represents a promising frontier in infectious disease research and clinical diagnostics. By leveraging advanced nanoparticle design, functionalization strategies, and diverse application routes, researchers can gain valuable insights into bacterial behavior, improve diagnostic accuracy, and develop targeted therapeutic approaches. As technology advances, the integration of nanoimaging into routine clinical practice may significantly enhance the management of infectious diseases. The pathogens listed in Table 4 represent significant public health concerns. For instance, *Escherichia coli* O157:H7 and *Salmonella typhimurium* are notorious for causing foodborne illnesses, making the application of quantum dots (Qdots) and magnetic nanoparticles (MNPs) in their detection crucial for food safety. Similarly, *Listeria monocytogenes* poses risks, particularly to vulnerable populations, and the use of MNPs with antibodies enhances detection capabilities in food products. *Mycobacterium tuberculosis*, the causative agent of tuberculosis, benefits from the sensitivity of dye-doped silica nanoparticles, which are essential for effective public health monitoring. Lastly, *Staphylococcus aureus*, a common pathogen responsible for various infections, is effectively detected using fluorescence-labeled polymeric nanoparticles and silicon nanoparticles, showcasing the versatility of these nanomaterials in clinical diagnostics. While nanomaterial-based pathogen detection shows clear promise across these examples, translating this preclinical potential into routine clinical practice raises several open questions, addressed below.

It is important to note that, despite more than a decade of preclinical promise for many of the nanomaterials summarized in Table 5, very few have progressed to clinical use, and this section would be incomplete without addressing why. Regulatory status is a major barrier: most of the nanoparticle platforms discussed above (quantum dots, magnetic nanoparticles, dye-doped silica nanoparticles) have not received regulatory approval for in vivo diagnostic use in humans, and remain restricted to in vitro, ex vivo, or food/environmental testing applications, which face a substantially lower approval bar than in vivo human diagnostics. Toxicity and biocompatibility present a further obstacle: heavy-metal-containing quantum dots (e.g., cadmium-selenide-based probes) raise long-term cytotoxicity and clearance concerns that have limited their progression beyond cell-culture and food-safety testing, and even biocompatible nanomaterials require extensive toxicology and biodistribution studies before first-in-human use. Manufacturing scalability and reproducibility are also limiting: many nanoparticle syntheses reported in the literature are optimized at laboratory scale with batch-to-batch variability in size, surface functionalization, and targeting-ligand density, which complicates the good manufacturing practice (GMP)-grade production required for clinical translation. Taken together, these translational barriers, rather than a lack of technical promise, largely explain the gap between the extensive preclinical nanoimaging literature summarized above and the very limited number of nanomaterial-based agents in clinical use or clinical trials for bacterial infection imaging.

To assist clinicians in evaluating the practical applicability of the tracers and radiopharmaceutical agents discussed above, Table 6 summarizes their reported infection-to-background ratio, typical imaging time window, and effective radiation dose, drawing on established nuclear medicine dosimetry references and the primary tracer-development studies cited throughout this review.

To address the wide range of evidence types that this review draws on from routinely available clinical modalities to early-phase human studies, animal models, and in vitro or food-safety detection assays we classify the techniques discussed throughout this review using the following evidence-level framework: (i) clinical/routinely available (approved, in routine clinical use); (ii) clinical research/human studies (used in humans but still investigational, e.g., early-phase trials or small case series); (iii) translational/large-animal studies; (iv) small-animal in vivo studies; (v) in vitro/ex vivo studies; and (vi) food/environmental pathogen detection (not intended for in vivo human diagnostic use). This classification is applied in Table 7 below and is noted qualitatively when introducing each of Table 2, Table 3, Table 4 and Table 5, to help readers distinguish established clinical capabilities from earlier-stage research findings that are presented alongside them. Table 7 synthesizes representative techniques from each modality class discussed in this review: anatomical/structural imaging, nonspecific and pathogen-specific functional imaging, and nanoimaging against sensitivity, specificity, spatial resolution, and the evidence-level/clinical-readiness classification introduced above, to give readers a comparative, at-a-glance context that is not evident from the individual case examples and study vignettes presented throughout this review (e.g., Figure 3).

## 4. Conclusions

**Figure 6:**
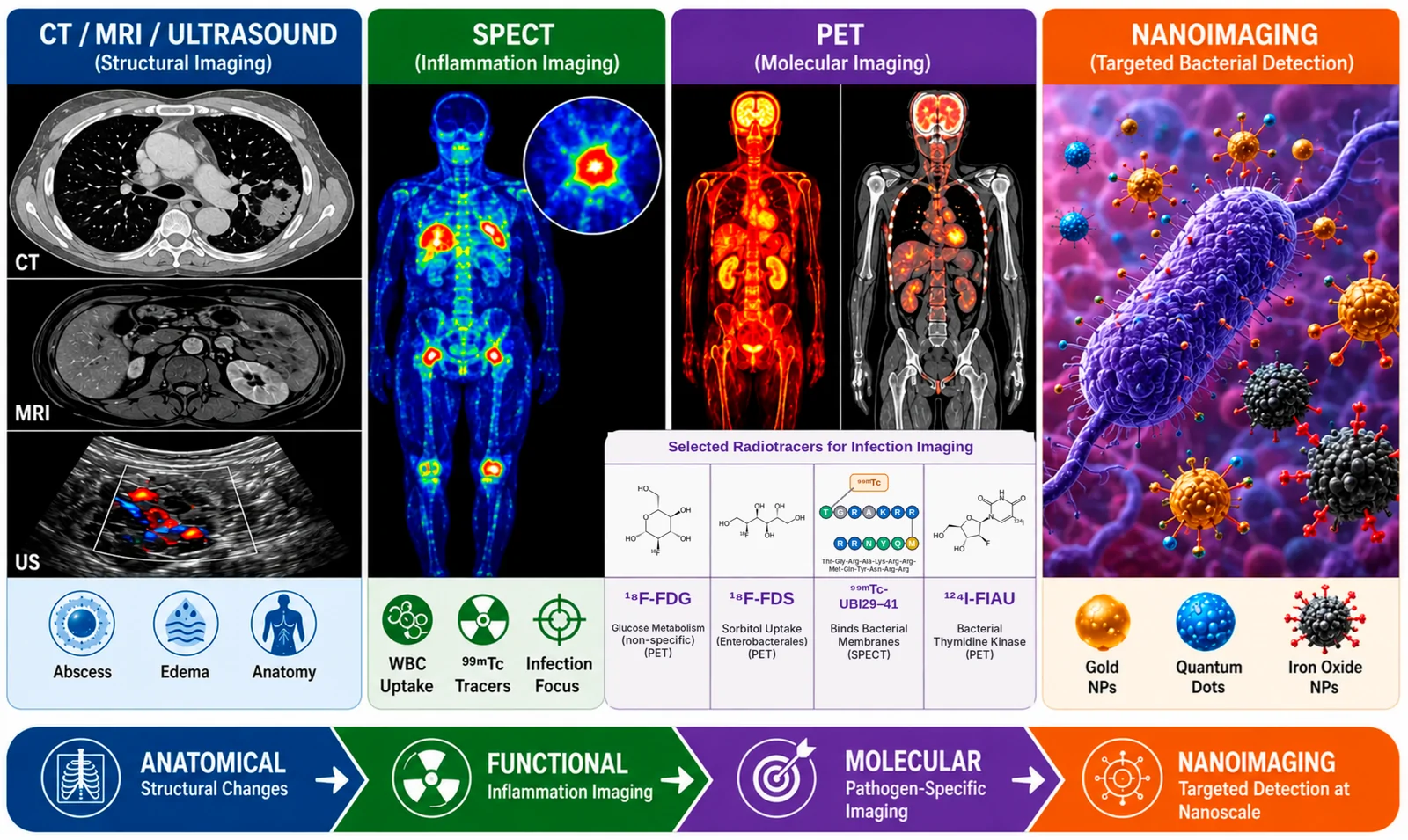
Schematic comparison of diagnostic methods for bacterial infection diagnosis and treatment monitoring. The background imaging panels were generated with a generative AI tool (Copilot) for illustrative purposes only and do not depict real patients. The tracer chemical structures and the UBI29–41 peptide sequence were drawn by the authors. ^18^F-FDG, ^18^F-FDS and ^124^I-FIAU are PET tracers; ^99m^Tc-UBI29–41 is a SPECT tracer [56,57,93,100].

Anatomical and functional imaging methods, along with nanoimaging techniques, play an established role in the investigation of infectious diseases and various inflammatory conditions. These modalities offer distinct advantages over conventional methods in determining disease activity, diagnosis, extent, and therapy monitoring. Strategies and recommendations for the utilization of anatomical, functional, and nanoimaging techniques are available for most of these applications. Despite the critical role of radiological imaging in detecting and localizing infections and inflammation, anatomical imaging alone is often insufficient for accurate diagnosis, as different diseases can present similar patterns. Therefore, functional imaging, which provides information about the functional activity of detected lesions, can significantly enhance the diagnostic process and response to treatment. Techniques such as single photon emission tomography (SPET), positron emission tomography (PET), computed tomography (CT), and magnetic resonance imaging (MRI) are highly sensitive for detecting functional or metabolic activity in infected or inflamed areas, allowing for effective monitoring of disease activity, particularly after treatment (Figure 6).

In recent years, nanoimaging methods have been extensively developed due to their exceptional advantages, including high sensitivity and cost-effectiveness. However, incidental findings from imaging can lead to unnecessary tests and treatments, increased radiation exposure, and additional costs; these drawbacks are significant concerns in infectious disease imaging. Moreover, when evaluating innovative tracers for imaging infections, it is crucial to consider various factors such as the infection model, the number and strain of pathogens involved, and the specific imaging settings. Additionally, the uptake of tracers in infected tissues should be correlated with tissue data and the viability of pathogens cultured from those tissues. Infectious diseases that lead to increased mortality are a significant public health issue. Early diagnosis and treatment are vital for managing acute bacterial infections, which can be challenging to treat effectively. Delays in diagnosis due to the slow growth of bacteria in culture can worsen patient outcomes. Traditional diagnostic methods, including microscopy and culture, have limitations, such as the need for specialized tools and personnel, as well as the potential for false positives and negatives. Therefore, increasing research in this area may enhance the utility of radiological evidence in diagnosing and treating microbial infections in the future. It should be acknowledged, however, that the translation of hybrid and nanoimaging approaches into routine clinical practice is constrained by several practical barriers that temper their otherwise promising outlook. Most pathogen-specific radiotracers and nanoimaging probes discussed in this review remain investigational and are available only at specialized academic centers with on-site cyclotron or radiopharmacy infrastructure, limiting real-world accessibility. Regulatory approval pathways for novel radiotracers are lengthy and costly, and require extensive toxicology, dosimetry, and clinical validation data before routine use can be considered, which has slowed the clinical translation of many otherwise promising agents. In addition, hybrid imaging equipment (PET/CT, SPECT/CT) and the radiopharmaceuticals themselves carry substantial acquisition and per-scan costs relative to conventional culture- and microscopy-based diagnostics, which may restrict their use in resource-limited settings despite their diagnostic advantages [145]. These considerations do not diminish the diagnostic value demonstrated in preclinical and early clinical studies but underscore that widespread adoption will depend on parallel progress in tracer manufacturing, cost reduction, and regulatory harmonization. The current review suggests that anatomical, functional, and nanoimaging approaches are convenient, effective, and increasingly safe for screening bacterial infections, monitoring disease progression, and guiding treatment decisions. These methodologies hold promising potential for clinical translation, provided the practical barriers outlined above are addressed.

## 5. Future Perspectives

Several converging developments are likely to shape the next generation of infection imaging over the coming years, extending well beyond incremental refinement of the techniques reviewed above.

***Artificial intelligence and deep learning:*** Machine learning algorithms are increasingly being integrated into PET and SPECT workflows for image reconstruction, denoising, automated lesion detection, and differentiation of infection from sterile inflammation or malignancy, with the potential to improve both the speed and consistency of interpretation across centers with varying levels of reader expertise [146]. Applying these tools specifically to infection-versus-inflammation classification, rather than the oncologic applications that have so far dominated the field, represents a promising but still underexplored direction.

***Total-body and ultra-sensitive PET:*** The recent clinical introduction of total-body PET/CT scanners, which offer a substantially larger axial field of view and higher sensitivity than conventional systems, enables whole-body infection screening with lower administered tracer activity and shorter acquisition times, and may improve detection of low-grade or multifocal infectious foci that are easily missed on regional imaging [147]. Broader clinical availability of this technology could meaningfully change infection imaging protocols, though cost and access remain limiting factors, as discussed in the Conclusion above.

***Next-generation pathogen-specific radiotracers:*** Beyond the tracers reviewed in Section 2, ongoing work continues to expand the repertoire of bacteria-specific probes, including radiolabelled siderophores, engineered antimicrobial–peptide hybrids designed to combine diagnostic and antibacterial activity, and refined folate- and sugar-pathway-targeted tracers progressing toward early-phase clinical evaluation [18,148]. Continued preclinical-to-clinical translation of these agents, supported by harmonized evaluation frameworks, will be necessary to realize their diagnostic potential in routine practice.

***Point-of-care and multiplexed platforms:*** Building on the nanoimaging and nanodiagnostic approaches discussed in Section 3 and the nano-based COVID-19 diagnostics noted in Section 2.1.2, portable and multiplexed biosensing platforms capable of combining pathogen detection with imaging-compatible signal output are an active area of development, with the potential to bring rapid, bedside infection diagnostics to settings that currently lack access to PET/SPECT infrastructure.

Taken together, these directions suggest that the field is moving toward more sensitive, more specific, and more accessible infection imaging. Realizing this potential, however, will require sustained investment in tracer development, imaging technology, computational tools, and resolution of the practical translational barriers outlined in the Conclusion above.

## Figures and Tables

**Table 1:** Applicable imaging techniques used clinically for diagnosis.

Techniques	Invasive/ Noninvasive	Working Principle	Disadvantages	Sample/ Target	Ref
X-ray	Noninvasive	An X-ray is created when a negatively stimulating electrode is heated by electricity, and electrons are released, thus producing energy. That energy is absorbed toward an anode, or metal plate, at high velocity, and an X-ray is produced when the energy hits the atoms in the metal plate.	X-rays involve exposure to ionizing radiation, which can increase cancer risk and is less effective for visualizing soft tissues, leading to potential misdiagnosis. They may produce false positives and negatives, causing unnecessary anxiety and follow-up imaging. Additionally, X-rays are generally avoided in pregnant patients due to risks to the fetus.	Bone, chest/lungs	[19,20]
Fluoroscopy	Noninvasive	Throughout a fluoroscopy technique, an X-ray beam is passed through the body. The image is transmitted to a monitor so that the movement of a body part, a tool, or a contrast agent (“X-ray dye”) allows the body to be seen in detail.	Fluoroscopy involves continuous exposure to ionizing radiation, increasing cancer risk, and providing limited soft tissue detail, which can lead to misinterpretation. It may require contrast agents that can cause allergic reactions and can be uncomfortable for patients. Additionally, the quality of images can be affected by patient movement, and the procedure can be costly and resource intensive.	Various (GI, vascular, musculoskeletal)	[21,22]
CT	Noninvasive	In the CT technique, a narrow beam of X-rays is aimed at a patient and rapidly rotated around the body, creating signals that are processed by the machine’s computer to produce cross-sectional images, or “slices.”	CT scans involve exposure to higher doses of ionizing radiation compared to other imaging modalities, increasing the risk of cancer over time. They may also produce artifacts or misinterpretations due to patient movement or overlapping structures, potentially leading to misdiagnosis. Additionally, the use of contrast agents in some CT scans can cause allergic reactions or kidney issues in susceptible individuals.	Whole-body/multiple organs	[23,24]
MRI	Noninvasive	A high-frequency electrical current is then pulsed through the patient, exciting the protons and causing them to become unbalanced and counteract the magnetic field’s attraction.	MRI can be expensive and time-consuming compared to other imaging modalities, which may limit accessibility for some patients. It is also contraindicated for individuals with certain implants or devices, such as pacemakers, due to the strong magnetic field. Additionally, some patients may experience anxiety or discomfort due to the enclosed space of the MRI machine and the need to remain still for extended periods.	Musculoskeletal, brain, soft tissue	[25,26]
Angiography	Invasive	Sensing contrast is injected into a blood vessel and projected on a series of X-rays to outline the inner vessel wall and show flow through the lumen.	Angiography involves exposure to ionizing radiation and the use of contrast agents, which can pose risks such as allergic reactions or kidney damage, particularly in patients with pre-existing renal conditions. The procedure is invasive, requiring catheter insertion, which carries risks of complications like bleeding or infection. Additionally, angiography may not always provide a complete picture of vascular conditions, potentially leading to misinterpretation or the need for further imaging.	Blood vessel	[27,28]
FKDS	Noninvasive	Ultrasound color Doppler combines anatomical information obtained using ultrasound pulse-echo technology with velocity information obtained using ultrasound Doppler technology to create grayscale images of tissue anatomy.	FKDS can be limited by its reliance on the availability of radiopharmaceuticals, which may not be accessible in all healthcare settings. The procedure may also involve exposure to radiation, albeit at lower levels compared to other imaging techniques, which can be a concern for some patients. Additionally, FKDS may have lower spatial resolution compared to other imaging modalities, potentially affecting the accuracy of kidney function assessment. [As a Doppler ultrasound technique, FKDS does not use radiopharmaceuticals or ionizing radiation; its actual limitations are that it is operator-dependent, has limited penetration depth in obese patients or deep structures, and flow-velocity measurements can be affected by the angle of insonation]	Tissue	[29,30]
Sonography	Noninvasive	The sound waves are reflected back to the transducer at boundaries between tissues in the beam path (between body fluids and soft tissue or between tissue and bone). When these echoes hit a transducer, they produce electrical signals that are sent to an ultrasound scanner.	It may also be operator-dependent, meaning the quality of the images and interpretations can vary based on the skill and experience of the technician. Additionally, while it is generally safe, there are concerns about the effects of prolonged exposure to ultrasound waves, particularly in sensitive populations like pregnant women.	Tissue and bone	[31,32]
IVUS	Invasive	The principle of IVUS imaging is based on the oscillatory movement (expansion and contraction) of a piezoelectric transducer (crystal) in order to yield sound waves while electrically excited.	IVUS is an invasive procedure that requires catheterization, which carries risks of complications such as bleeding, infection, or vascular injury. The interpretation of IVUS images can be complex and may require specialized training, potentially leading to variability in results. Additionally, IVUS may not provide comprehensive information about the surrounding tissue or blood flow dynamics, limiting its diagnostic capabilities compared to other imaging modalities.	Tissue	[33,34]
SPECT	Noninvasive	SPECT involves injecting a small amount of a radioactive tracer into the bloodstream. Inside the body, tracers produce a type of energy called gamma rays. A gamma-ray camera records the tracer signal, and a computer converts it into an image of blood flow through the heart.	SPECT involves exposure to ionizing radiation, which can pose a risk, particularly with repeated scans over time. The spatial resolution of SPECT is generally lower than that of other imaging techniques, such as PET or MRI, which may affect diagnostic accuracy. Additionally, SPECT requires the use of radiopharmaceuticals, which can lead to allergic reactions or other side effects in some patients.	Tissue	[35,36]
PET	Noninvasive	PET mechanism uses a scanning device to sense photons (subatomic particles) emitted by a radionuclide in the organ or tissue being observed.	PET involves exposure to ionizing radiation, which can increase cancer risk, especially with repeated scans. The procedure can be expensive and may not be widely available in all healthcare settings, limiting accessibility for some patients. Additionally, PET scans often require the use of radiotracers, which can cause allergic reactions or other side effects in certain individuals.	Whole-body (metabolically active tissue)	[37,38]
NIRF	Invasive	NIRF works on a principle of specimen scanning in a raster mode of 1.0–1.5 mm with a lower spatial resolution	NIRF imaging has limitations in in-depth penetration, making it less effective for visualizing deeper tissues compared to other imaging modalities. The technique can also be affected by tissue absorption and scattering, which may reduce image quality and accuracy. Additionally, NIRF requires the use of fluorescent dyes, which can pose risks of allergic reactions or toxicity in some patients.	Blood	[39,40]
NIRS-IVUS	Invasive	Work-based on the relative transparency of tissue to light in the NIR range and the oxygenation-dependent light-absorbing features of hemoglobin	NIRS-IVUS combines two imaging modalities, but it can be limited by the complexity of the procedure, requiring specialized training and expertise for accurate interpretation. The technique may also face challenges with signal interference from surrounding tissues, potentially affecting the quality of the data obtained. Additionally, as an invasive procedure, it carries risks associated with catheterization, such as bleeding, infection, or vascular injury.	Blood, tissue	[41,42]

Intravascular ultrasound (**IVUS**), color-coded duplex sonography (**FKDS**), near-infrared spectroscopy (**NIRS-IVUS**), near-infrared fluorescence (**NIRF**).

**Table 2:** SPECT tracers for specific detection of infections, including their targets, mechanisms, and examples.

Tracer Type	Target	Mechanism of Action	Examples	Ref
Cell Wall Targeting Tracers **	Bacterial cell wall components	Bind specifically to peptidoglycan or other cell wall structures	Radiolabeled antibodies, peptides	[48]
**Metabolic Activity Tracers**	Metabolically active cells	Reflect increased metabolic activity in infected tissues	^18^F-FDG (fluorodeoxyglucose)	[50]
**Virulence Factor Targeting**	Pathogen-specific virulence factors	Bind to toxins or enzymes indicative of pathogenicity	Radiolabeled antibodies to toxins	[54]
**UBI Peptides**	Bacterial cell wall components	Engineered to bind specifically, enhancing imaging specificity	UBI peptide derivatives	[56,57]
**AMPs**	Bacterial membranes and structures	Possess antimicrobial properties while providing imaging capabilities	LL-37, defensins	[56,57]

** Cell Wall Targeting Tracers: These tracers are particularly useful for identifying bacterial infections by targeting unique components of the bacterial cell wall**. Metabolic Activity Tracers**: These tracers are beneficial for assessing the viability of pathogens based on their metabolic activity, which is often elevated during infection. **Virulence Factor Targeting**: This approach helps differentiate between pathogenic and non-pathogenic strains, providing insights into the severity of the infection. **UBI Peptides and AMPs**: These novel tracers not only aid in imaging but also have therapeutic potential due to their antimicrobial properties. This table provides a concise overview of the various SPECT tracers used for infection detection, highlighting their specific targets and mechanisms of action.

**Table 3:** Anatomical or morphological imaging in bacterial infection.

Infection	Bacteria	Methods	Target	Ref
Pyogenic	*S. pyogenes*	CT	Lung parenchyma	[76,77,78]
_	*S. aureus*	Raman-based imaging techniques	Endothelial cells	[79]
CAP	Not pathogen-specific	Chest radiography	PCT and hsCRP	[80]
Pulmonary	Pulmonary bacterial secondary infections and co-infections with SARS-CoV-2 are uncommon.	CT scan	Pulmonary	[81]
Skin and soft tissue	Complex microorganisms	CT scan	Skin and soft tissue	[82]
Myositis	*S. aureus*	MRI, GFV	Myositis	[83]
Meningitis	*N. meningitides*, *S. pneumoniae*	MRI	Brain	[84]
Spondylodiscitis	Tuberculous and other microorganisms	MRI	The intervertebral disk and the adjacent vertebrae	[85]
Musculoskeletal	Complex microorganisms	MRI	Joint, soft tissue, and bone	[86]
SSTIs	*S. aureus*	Ultrasound	Skin and soft tissue	[70]
Cholecystitis	*Escherichia coli*, *Klebsiella*, and *Streptococcus faecalis*	US	Gallbladder	[87]
PJI	*S. aureus*, *E. faecalis*, *E. coli*, *K. pneumoniae*	US	Joint	[88]
PTA	*Streptococcus pyogenes*, *F. necrophorum*, and *F. nucleatum*	MRI, US	Peritonsillar space	[89]

Skin and soft tissue infections (**SSTIs**); ultrasound (**US**); periprosthetic joint infection [**PJI**]; peritonsillar abscess (**PTA**).

**Table 4:** Some details of functional imaging methods in the diagnosis of bacterial infections.

Infection Type	Bacteria	Radiolabels/Data	Imaging Technique	Target	Ref
Musculoskeletal	_	^18^F-FDG PET	PET scan, SPECT	_	[123,124]
Musculoskeletal	*Various bacteria*	[^124^I]FIAU	PET/CT	TK	[93]
_	*P. aeruginosa* (strain PAO1)	CDy11	Fluorescent probe	Amyloid	[98]
Pyogenic	*S. pyogenes*	^111^In-labeled DOTA-biotin and Zn-DPA-biotin	SPECT-CT	_	[99]
*Enterobacterales*	*Enterobacteria*	2-deoxy-2-[^18^F]fluoro-d-sorbitol (^18^F-FDS)	PET	Infection	[100]
*Enterobacterales*	*Enterobacteria*	^18^F-FDS	PET/CT	Infection	[101]
CIED	*S. aureus*	2-[^18^F]fluoro-2-deoxy-*D*-glucose (^18^F-FDG)	PET/CT	Cardiac	[102]
Co-infections	Complex microorganisms	^18^F-FDG	FDG PET/CT	Blood cells	[103]
VGEI	Complex microorganisms	^18^F-FDG PET/CT	FDG PET/CT	Vascular graft and endograft	[125]
Localized	*S. aureus* (strain Xen29)	2-[^18^F] F-ENB and 2-[^18^F] F-NB	PET/CT	Bacteria	[106]
Pulmonary	*M. tuberculosis*	^18^F-FDG	PET/CT	Bacteria	[107]
Unknown	_	^18^F-FDG	FDG-PET/CT	Unknown	[108]
Metastasis	Complex microorganisms	^18^F-FDG	FDG-PET/CT	Lung/Pelvic	[109]
Endocardial	*Pneumococci*	^18^F-FDG	FDG-PET/CT	Pulmonary	[112]
Vascular graft	*Escherichia coli*	^18^F-FDG	^18^F-FDG PET/CT	Vascular	[115]
Spondylodiscitis	*S. aureus*	^18^F-FDG	^18^F-FDG PET/CT	Spine	[115]
Cyst Infection	*Escherichia coli*	^18^F-FDG	^18^F-FDG PET/CT	Chest	[115]
Environmental pollutants and biological molecules	Recombinant luminescent bacteria	^NA^	^NA^	Chest	[117]
Otitis media-causing bacteria	*Haemophilus influenzae*, *Streptococcus pneumoniae*, *Moraxella catarrhalis*	^NA^	^NA^	Middle ear	[118]

Thymidine kinase (**TK**); community-acquired pneumonia (**CAP**); C-reactive protein (**hsCRP**); procalcitonin (**PCT**); cardiac implantable electronic device (**CIED**); vascular graft and endograft infection (**VGEI**). Bacterial identifications throughout this review are presented at the taxonomic resolution reported in each cited primary source. Where the original study specified an exact strain or isolate, we have now added this detail (e.g., *P. aeruginosa* strain PAO1 and *S. aureus* strain Xen29, Table 4; *E. coli* O157:H7, *S. typhimurium*, and *L. monocytogenes*, Table 5). For most preclinical PET/SPECT tracer studies and, in particular, the clinical case reports underlying Table 4, the source publications identified pathogens only at the species level (e.g., *S. aureus*, *M. tuberculosis*) without a specific ATCC reference number or clinical isolate designation, reflecting standard clinical microbiology reporting rather than an omission on our part. It has not inferred or assigned strain identifiers that were not reported in the original source, as doing so would risk misrepresenting the primary literature. Readers seeking strain-level detail to evaluate the specificity claims of a given tracer are encouraged to consult the cited primary reference directly.

**Table 6:** Comparative overview of key radiotracers and cell-labeling agents used for infection imaging, including infection-to-background ratio, imaging time window, and effective radiation dose.

Tracer/Agent (Modality)	Infection-to-Background Ratio	Typical Time-to-Image (Post-Injection)	Effective Radiation Dose (Adult, Standard Activity)	Ref
^18^F-FDG (PET/CT)	High, rapid, high-magnitude uptake at infection foci relative to background	~60–90 min	~7 mSv (370–555 MBq)	[108,115]
^99m^Tc-HMPAO-labeled leukocytes (SPECT)	High, regarded as a reference standard for infection localization	Early images 1–4 h; delayed images up to 20–24 h	~2–6 mSv (185–370 MBq)	[144]
111In-oxine-labeled leukocytes (SPECT)	High, but slower kinetics than ^99m^Tc-WBC	Delayed imaging at 4 h and 18–24 h (occasionally to 48 h)	~7–11 mSv (18–37 MBq)	[144]
67Ga-citrate (planar/SPECT)	Moderate–high but nonspecific (also accumulates in tumors and sterile inflammation)	Delayed imaging, typically 48–72 h post-injection	~15–22 mSv (typical adult activity)	[144]
2-[^18^F]F-PABA (PET, preclinical)	~8-fold infection-to-background differentiation vs. sterile inflammation (rodent model)	Rapid; rodent imaging within ~1 h post-injection	Not yet established in humans (preclinical only)	[106]
2-[^18^F]F-ENB/2-[^18^F]F-NB (PET, preclinical)	~17-fold infection-to-background differentiation (rodent model); improved over 2-[^18^F]F-PABA	Rapid; rodent imaging within ~1 h post-injection	Not yet established in humans (preclinical only)	[106]
UBI peptides/AMP-based tracers	Variable; differentiate infection from sterile inflammation in preclinical/early clinical studies, though less standardized than FDG or WBC scintigraphy	Typically 30–60 min	Not uniformly established across tracer variants	[56,57]

Values represent typical or reported ranges compiled from the cited sources and may vary with administered activity, patient body habitus, and scanner/camera characteristics. “Not yet established in humans (preclinical only)” indicates that, to our knowledge, no validated human dosimetry data have been published for that agent at the time of writing. UBI, ubiquicidin; AMP, antimicrobial peptide; WBC, white blood cell; HMPAO, hexamethylpropyleneamine oxime; PABA, para-aminobenzoic acid.

**Table 7:** Comparative sensitivity, specificity, spatial resolution, and clinical readiness across representative anatomical, functional, and nanoimaging techniques for bacterial infection imaging.

Modality Class	Representative Technique(s)	Sensitivity	Specificity	Spatial Resolution	Clinical Readiness (per Comment 2 Classification)
Anatomical/structural imaging	CT, MRI, ultrasound, X-ray	High for late-stage structural change (e.g., abscess, bone destruction); low for early/subclinical infection	Low–moderate; cannot reliably distinguish infection from sterile inflammation, tumor, or postsurgical change	Sub-mm to ~1 mm (CT, MRI); operator- and probe-dependent for ultrasound	Clinical/routinely available–approved, in widespread routine use
Functional imaging, nonspecific tracer	^18^F-FDG PET/CT	~86–95% (pooled, varies by indication) [108]	~89–92% (pooled, varies by indication) [108]	~4–6 mm (PET component)	Clinical/routinely available–approved, in widespread routine use
Functional imaging, nonspecific tracer	^99m^Tc-HMPAO/111In-oxine labeled leukocyte scintigraphy	~80–90% (pooled, condition-dependent)	~85–100% (pooled, condition-dependent); regarded as a reference standard	~1 cm (planar/SPECT)	Clinical/routinely available–approved, in widespread routine use
Functional imaging, pathogen-specific tracer	UBI(29-41) and derivatives (SPECT/PET)	Reported high in small clinical studies, but not yet established by large-scale meta-analysis	Reported high in small clinical studies (differentiates infection from sterile inflammation), not yet established by large-scale meta-analysis	~1 cm (SPECT); improved with PET-labeled analogs	Clinical research/human studies–early-phase clinical trials, not yet routinely available
Functional imaging, pathogen-specific tracer	2-[^18^F]F-PABA, 2-[^18^F]F-ENB/F-NB, ^18^F-FDS	High infection-to-background differentiation in rodent models (see Table 6); limited human data	High in preclinical models; human specificity data are still limited	~1–2 mm (small-animal PET)	Small-animal in vivo to early clinical–most human data still limited to case series
Nanoimaging (fluorescent/quantum dot probes)	Quantum dots, dye-doped silica NPs, magnetic NPs (Table 5)	High analytical sensitivity in vitro (down to single-cell/low-CFU detection)	Depends on targeting ligand specificity; not systematically validated in vivo	Sub-micron (fluorescence microscopy, in vitro)	In vitro/ex vivo and food/environmental detection–not yet tested in vivo in humans.
Nanoimaging (amyloid/biofilm-targeting probes)	CDy11 and related fluorescent probes	High in preclinical biofilm models	High for amyloid-associated biofilm structures in preclinical models	Sub-micron (fluorescence imaging)	Small-animal in vivo–not yet in human trials

Sensitivity and specificity ranges reflect pooled or representative values reported in the cited meta-analyses and primary studies referenced throughout this review; values vary by clinical indication, patient population, and reference standard used, and single-study or small-series values may differ substantially from pooled estimates. Where large-scale human diagnostic-accuracy data are not yet available (most pathogen-specific tracers and all nanoimaging agents), this is stated explicitly rather than estimated.

## Data Availability

No new data were created or analyzed in this study. Data sharing is not applicable to this article.
